# Comparative Assessment of Protein Kinase Inhibitors in Public Databases and in PKIDB

**DOI:** 10.3390/molecules25143226

**Published:** 2020-07-15

**Authors:** Colin Bournez, Fabrice Carles, Gautier Peyrat, Samia Aci-Sèche, Stéphane Bourg, Christophe Meyer, Pascal Bonnet

**Affiliations:** 1 Institut de Chimie Organique et Analytique (ICOA), UMR CNRS-Université d’Orléans 7311, Université d’Orléans BP 6759, 45067 Orléans CEDEX 2, France; colin.bournez@univ-orleans.fr (C.B.); fabrice.carles@univ-orleans.fr (F.C.); gautier.peyrat@univ-orleans.fr (G.P.); samia.aci-seche@univ-orleans.fr (S.A.-S.); stephane.bourg@univ-orleans.fr (S.B.); 2 Janssen-Cilag, Centre de Recherche Pharma, CS10615-Chaussée du Vexin, 27106 Val-de-Reuil, France; cmeyer2@its.jnj.com

**Keywords:** protein kinase inhibitors, clinical trials, approved drugs, database, chemometrics analysis, kinome, molecular scaffolds, rings system

## Abstract

Since the first approval of a protein kinase inhibitor (PKI) by the Food and Drug Administration (FDA) in 2001, 55 new PKIs have reached the market, and many inhibitors are currently being evaluated in clinical trials. This is a clear indication that protein kinases still represent major drug targets for the pharmaceutical industry. In a previous work, we have introduced PKIDB, a publicly available database, gathering PKIs that have already been approved (Phase 4), as well as those currently in clinical trials (Phases 0 to 3). This database is updated frequently, and an analysis of the new data is presented here. In addition, we compared the set of PKIs present in PKIDB with the PKIs in early preclinical studies found in ChEMBL, the largest publicly available chemical database. For each dataset, the distribution of physicochemical descriptors related to drug-likeness is presented. From these results, updated guidelines to prioritize compounds for targeting protein kinases are proposed. The results of a principal component analysis (PCA) show that the PKIDB dataset is fully encompassed within all PKIs found in the public database. This observation is reinforced by a principal moments of inertia (PMI) analysis of all molecules. Interestingly, we notice that PKIs in clinical trials tend to explore new 3D chemical space. While a great majority of PKIs is located on the area of “flatland”, we find few compounds exploring the 3D structural space. Finally, a scaffold diversity analysis of the two datasets, based on frequency counts was performed. The results give insight into the chemical space of PKIs, and can guide researchers to reach out new unexplored areas. PKIDB is freely accessible from the following website: http://www.icoa.fr/pkidb.

## 1. Introduction

**Figure 1:**
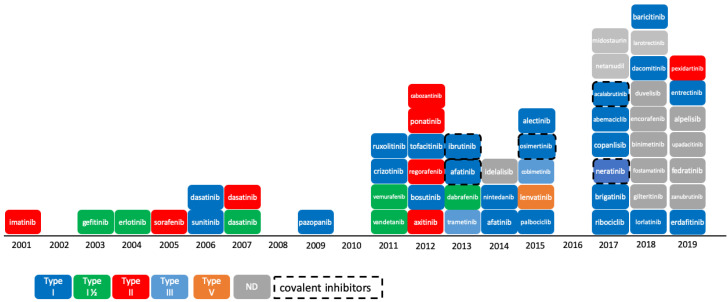
Progression of Food and Drug Administration (FDA)-approved protein kinase inhibitors (2001–2019) and their type of inhibition [12]. As of 11th December 2019, 55 kinase inhibitors were approved by the FDA. Not shown here: tivozanib, approved by European Medicines Agency (EMA) in 2017; anlotinib, apatinib, and icotinib, approved by the China Food and Drug Administration (CFDA) in 2018, 2014, and 2011, respectively; and fasudil, approved in China and in Japan in 1995. ND: not defined.

The reversible phosphorylation of proteins plays a preeminent role in cell cycle regulation. This process, which consists in the transfer of a phosphoryl group PO_3_^2−^ to the target substrate, is catalyzed by enzymes pertaining to the protein kinase family. Protein kinases constitute one of the largest protein families encoded by the human genome and counts 518 members (or 538 members when atypical kinases are included) [1,2,3]. Numerous studies have shown that deregulation or mutation of kinases is responsible for a variety of cancers [4], as well as for other diseases in the immune or neurological area [5,6]. The majority of protein kinases, however, have not yet been fully explored [7], and there is still a high potential for innovation in targeting the protein kinome for the treatment of cancer. The Food and Drug Administration (FDA) approved 55 small-molecule protein kinase inhibitors (PKIs) by end of 2019, whereas the Chinese and European regulatory authorities have granted market access to five more compounds, namely anlotinib, apatinib, icotinib, fasudil, and tivozanib (Figure 1). It is worth mentioning that higher molecular weight inhibitors like macrocyclic lactones, such as sirolimus and temsirolimus, or kinase-targeted antibodies, such as cetuximab and trastuzumab, have been approved for the treatment of colorectal, head/neck, and breast cancers, respectively [8,9,10]. These large molecules were excluded from this study, which focuses on small-molecule PKIs targeting the kinase domain. The first PKI approved by the FDA was imatinib in 2001. Imatinib is a small-molecule type-II inhibitor containing a phenylamino-pyrimidine scaffold. It targets the inactive conformation of ABL1 kinase and is used against chronic myelogenous leukemia (CML) [11]. Since then, at least one new PKI reaches the market every year, with a significant acceleration since 2011. The exceptions to this rule are 2002, 2008, 2010, and 2016, with no compound approved in these years.

In addition to approved PKIs, many novel compounds are currently being evaluated in clinical trials throughout the pharmaceutical industry. Taken collectively, these compounds show new trends in terms of structures, physicochemical properties, and biological activities that foreshadows changes in the PKI landscape. To collect and organize this data as well as keep up to date with their evolution, we developed PKIDB [12], a curated, annotated and updated database of PKIs in clinical trials. In order to enter the PKIDB, compounds should be currently in one development phase (from Phase 0 to Phase 4), have a disclosed chemical structure, as well as an International Nonproprietary Name (INN) [13]. Each compound is provided with comprehensive descriptive data, as well as with links to external databases such as ChEMBL [14], PDB [15], PubChem [16], and others. The type of binding mode specified in PKIDB has been manually entered and comply with Roskoski’s classification [12]. The database is freely accessible on a dedicated website (http://www.icoa.fr/pkidb). As of 11th of December 2019, it contains 218 inhibitors: 60 approved and 158 in various stages of clinical trials (from Phase 0 to Phase 3).

In this study, we compared PKIDB to a large dataset of 76,504 PKIs retrieved from ChEMBL (referred herein as “PKI_ChEMBL”, see the Materials and Methods section). The objective is to be able to better select PKIs from public databases based on structural and physicochemical property information of PKIs already in clinical trials. Firstly, we performed a principal component analysis (PCA), and compared the projection of both datasets in a common factorial space. We also assessed the structural shape diversity of PKIs using a principal moments of inertia (PMI) analysis. Secondly, in addition to comparisons at the global molecular structure level, we performed a substructure analysis based on PKI scaffolds. In medicinal chemistry, scaffolds are mostly used to represent core structures of bioactive compounds. They are relevant for the medicinal and/or computational chemists, and have proved to be useful in the identification of “privileged substructures” [17] in “scaffold hopping” [18] or in structure–activity relationships (SAR) analyses [19]. The concept of the scaffold was first defined by Bemis and Murcko, as frameworks that consist of rings and linkers, from which substituents are removed [20]. From these scaffolds, two levels of abstraction were derived: the heteroatom framework and the graph representation. The heteroatom framework only takes into account the atom type, without considering bond types or aromaticity, whereas the graph representation (also known as cyclic skeleton) turns every atom type to carbon and every bond type to a single bond, reducing the initial molecule to a simple graph [21]. Finally, unfused rings present in the molecules are separated by removing their connecting bonds.

The balance between the molecular diversity of scaffolds, and their frequency is an important parameter in a chemical database. A high frequency associated to a small number of scaffolds corresponds to a focused library composed of structurally similar molecules, bearing varying substituents. Contrarily, a low frequency associated to a large number of scaffolds reflects a high molecular diversity [12]. Thus, this criterion needs to be addressed when designing or selecting a chemical library depending on its forecasted usage. We assessed scaffold diversity for the PKIDB and PKI_ChEMBL datasets using the molecular Bemis and Murcko scaffolds and cyclic skeleton. The most represented scaffolds (frequency) and the comparison of their distribution within the two studied datasets are presented. Finally, an analysis of the rings of all molecules was performed. We first considered all the rings devoid of substituent (first attached atoms were replaced by hydrogen atoms). Then, we encoded the rings while retaining the position and atom type of their original substituents. This scaffold diversity analysis reflects the chemical space of PKIs and can be useful for the medicinal chemistry community to reach out new unexplored areas.

## 2. Results

### 2.1. Update on PKIDB

The description and analysis of PKIDB have been reported in a previous study by Carles et al. [22]. Referencing 218 molecules the 11th December 2019, PKIDB contains 38 more inhibitors (from phase 0 to phase 4) than the first release (abivertinib, adavosertib, alvocidib, asciminib, avapritinib, bemcentinib, berzosertib, bimiralisib, capivasertib, ceralasertib, derazantinib, dezapelisib, enzastaurin, fasudil, lazertinib, leniolisib, mavelertinib, midostaurin, nazartinib, neflamapimod, nemiralisib, netarsudil, ningetinib, parsaclisib, pralsetinib, ravoxertinib, ripasudil, ripretinib, rivoceranib, rogaratinib, ruboxistaurin, samotolisib, sotrastaurin, tomivosertib, umbralisib, vactosertib, verosudil, zanubrutinib).

Among these 38 compounds, nine were FDA-approved in 2017, eight in 2018, and seven in 2019. Fasudil, a ROCK inhibitor, approved in China and in Japan in 1995 was therefore the first kinase inhibitor that reached the market, but it is not FDA approved. Those compounds were automatically added to PKIDB database thanks to their name stem. Indeed, since the first release of PKIDB, the INN made an update on the stems that assign the molecules with the “aurin” and “udil” suffixes to the kinase inhibitor class. Moreover, the stem ‘cidib’ was also updated and has been replaced by ‘ciclib’ (see cumulative USAM stem list from AMA [23]). However, we also kept the stem ‘cidib’ to retrieve information on alvocidib, not yet referenced as alvociclib.

In addition to those compounds, Table 1 gathers the eight and seven PKIs that reached phase 4 and were FDA-approved in 2018 and 2019, respectively. Among those 15 PKIs, all were previously in a phase lower than 4 in our database, except zanubrutinib, which was not in the first release. One should note that FDA recently approved avapritinib, a selective dual KIT and PDGFRα inhibitor, after the updated version of PKIDB, and is therefore not considered in this study.

This brings the total number of approved drugs on the market referenced in our database to 60. As described in PKIDB, most of the PKIs are targeting more than one protein kinases, and since the first version of PKIDB, new targets have emerged, such as the Wee1-like protein kinase inhibited by adavosertib [24].

### 2.2. Physicochemical Analysis of PKI Datasets

#### 2.2.1. Distribution of Physicochemical Properties of PKIs

**Figure 2:**
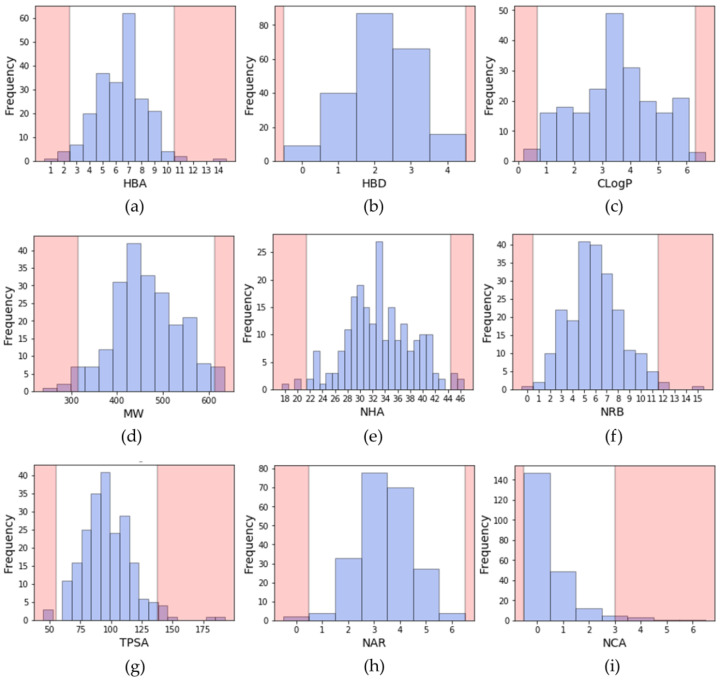
Distribution of physicochemical properties of PKIs: (**a**) number of hydrogen bond acceptors (HBA); (**b**) number of hydrogen bond donors (HBD); (**c**) ClogP (Rational Discovery Kit (RDKit)); (**d**) molecular weight (MW); (**e**) number of heavy atoms (NHA); (**f**) number of rotatable bonds (NRB); (**g**) topological polar surface area (TPSA); (**h**) number of aromatic rings (NAR); (**i**) number of chiral atoms (NCA). Pink areas represent values outside two standard deviation from the mean (95.4% confidence interval).

To describe a molecule, it is common to compute its physicochemical properties to obtain information on the size, the lipophilicity, the atomic composition, etc. Some descriptors, as described by Lipinski or Veber, are still widely used to evaluate the potential oral bioavailability of a compound [25,26]. Lipinski rule relies on four properties: molecular weight (MW) ≤ 500; number of hydrogen bond donors (HBD) ≤ 5; number of hydrogen bond donors (HBA) ≤ 10 and logP ≤ 5. Veber rule relies on the number of rotatable bonds (NRB) ≤ 10 and topological polar surface area (TPSA) ≤ 140 Å or the sum of HBD and HBA ≤ 12. In addition, since they are also important in drug design, the number of aromatic rings and the number of chiral atoms were also calculated [27,28]. During the search of a lead compound in a virtual screening campaign, such descriptors may serve as a filter to discard molecules, and therefore decrease the size of the chemical library, since a virtual library can be large. The distribution of these descriptors calculated from inhibitors extracted from PKIDB is shown in Figure 2.

In a previous study [22], we analyzed the ‘rule of five’ descriptors detailed by Lipinski [25] for inhibitors in clinical trials and approved. Here, we updated the statistical analysis with new PKIs included in PKIDB and we compared them to the ChEMBL dataset (Table 2).

We found that 62% and 68% of PKIs in PKIDB and in ChEMBL, respectively, do not violate any Lipinki’s rule. One single violation occurs in 28% and 24% of the PKIs for PKIDB and ChEMBL, respectively, and two violations occur for about 10% of the PKIs in the two datasets. Finally, few PKIs in ChEMBL dataset violates more than two rules (0.2%), and none for the PKIs in PKIDB. These results may vary, depending on how the LogP is calculated. Here, we used Wildman-Crippen approach [29]. Compared to the initial study, we removed the counter ion during the standardization of the molecules, such as the bromide ion in tarloxotinib. Despite the large different number of compounds in both datasets (76,504 molecules in ChEMBL and 218 in PKIDB) we revealed that the two datasets exhibit similar rule of five violation profiles.

The ratio of PKIs having descriptors out of the Lipinski’s or Veber’s rule are given in Table 3. Here, again, we found that there is no significant difference between the two kinase subsets over all the descriptors. Molecular weight (MW) and CLogP are the most discriminant descriptors. Interestingly, less than 5% of the PKIs have descriptors out of Veber’s boundaries.

From these calculations, we propose a range of descriptors to guide the design of kinase inhibitors. The proposed ranges do not consider the property values beyond two standard deviations from the mean (95.4% confidence interval). Thus, the upper and lower limits of molecular descriptors better represent the current chemical space of kinase inhibitors, either approved or in clinical trials.

One can notice that despite new PKIs in PKIDB, these guidelines have not changed much compared to the ones presented in our first study. This shows that the define PKI chemical space seems well defined.

Considering all PKIs from PKIDB, the guidelines for prioritization are:

- A molecular weight (MW) between 314 and 613 Da (average of 463.4 Da);
- A ClogP (calculated with a Rational Discovery Kit (RDKit)) between 0.7 and 6.3 (average of 3.5);
- Between 0 and 4 hydrogen bond donors (HBD) (average of 2.2);
- Between 3 and 10 hydrogen bond acceptors (HBA) (average of 6.4);
- A topological polar surface area (TPSA) comprised between 55 and 138 Å^2^ (average of 96.6 Å^2^);
- Between 1 and 11 rotatable bonds (NRB) (average of 6.0);
- Number of aromatic rings (NAR) between 1 and 5 (average of 3.4);
- Number of chiral atoms (NCA) between 0 and 2 (average of 0.5).

#### 2.2.2. Statistical Analysis of Protein Kinase Inhibitors

To compare the chemical space of the kinase inhibitors from PKIDB and from ChEMBL (PKI_ChEMBL), we performed a principal component analysis (PCA). Each molecule was described using 10 classical physicochemical descriptors (See Materials and Methods) well suited to characterize chemical structures. The goal here is to compare PKI_ChEMBL to PKIDB.

**Figure 3:**
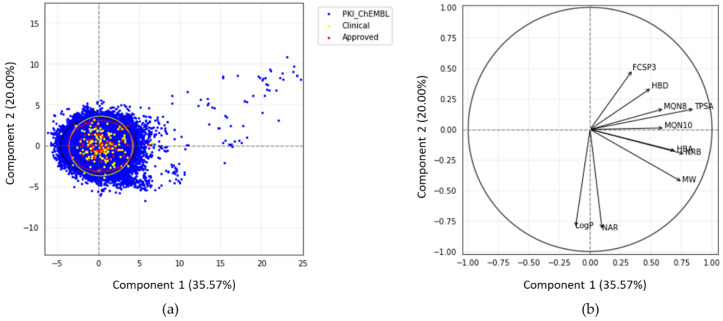
(**a**) Principal component analysis (PCA) of PKIs from ChEMBL and PKIDB, containing 76,504 and 209 compounds, respectively. Black, yellow, and red ellipses encompass 95% of the individuals from class “PKI_ChEMBL”, “Clinical_PKI”, and “Approved_PKI”, respectively; (**b**) correlation circle.

The PCA plot (Figure 3) illustrates the chemical space of PKIs in a 2D reference frame, represented by the two first principal components (PC1 and PC2).

The two first principal components explain 35.6% and 20.0% of the total variance respectively. PC3, not shown here, encompasses 13.2%. Thus, the 2D scatterplot of the factorial space illustrated here represents around 56% of the total variance (Figure 3).

Each dot on the graph (Figure 3a) represents a molecule. Few compounds from PKI_ChEMBL are projected in the upper right quadrant but none belongs to PKIDB. Most of the PKIDB compounds are centered in the PCA plot. Approved (red dots) and in clinical trials (yellow dots) PKIs are projected in the same chemical space. The graphical representation of normalized variables is shown in the correlation circle (Figure 3b). The angle between two vectors indicates the correlation between the two corresponding variables. A value close to 0° or 180° indicates positively or negatively correlated variables, respectively. A value near 90° indicates that the variables are not correlated. On the correlation circle (Figure 3b), one can see that the first factorial axis (PC1) is highly correlated with TPSA, NRB, and MW. These three variables contribute to PC1 at 20.6%, 17.1%, and 16.1%. The variables CLogP and NAR do not contribute to this axis, and are negatively correlated with the second factorial axis (PC2). Their contribution to PC2 are 32.6% and 34.0%, respectively. To a lesser extent, this axis is also positively correlated with FCSP3 and HBD (contributions of 11.8% and 5.8%, respectively). These two descriptors are correlated to PC3 (contributions of 24.7% and 29.7%, respectively). A weak angle between NAR and CLogP vectors is consistent with the fact that CLogP increases with the number of aromatic rings.

In view of these results, PCA confirms our preliminary observations that there are few outliers in PKI_ChEMBL dataset (dots on the upper right quadrant). It appears that these compounds correspond to either small-modified peptides or macrocyclic lactones with high TPSA values. These molecules, such as everolimus, were removed from PKIDB, since they do not inhibit protein kinases directly, however, the macrocycles in PKI_ChEMBL are active on protein kinases and, thus, were not removed from the dataset. Regarding the compounds in PKIDB, semaxanib, has the lowest MW (yellow dot, bottom-left). The two dots outside the circle and on the middle right of the quadrant corresponds to barasertib (Clinical_PKI in yellow) and fostamatinib (Approved_PKI in red). Both of these molecules contain phosphate group, increasing their TPSA, and therefore explaining their position on the PCA map.

#### 2.2.3. Principal Moments of Inertia

Until now, we only analyzed the molecules using 2D descriptors; therefore, to evaluate the shape diversity, we represented the molecules on a principal moments of inertia (PMI) plot [30]. In a triangular PMI map, the three corners represent distinctive shapes: rod (represented by diacetylene), disk (benzene) and sphere (adamantane). Note that such a plot only describes molecular shapes, without any consideration of other properties. In order to escape from the flatland, compounds should get closer to the sphere [31].

**Figure 4:**
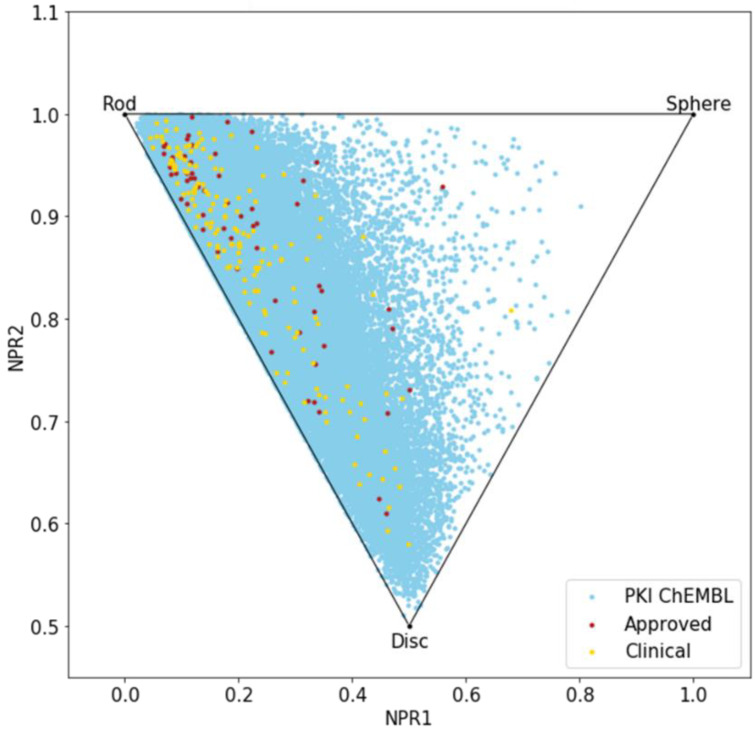
Principal moments of inertia (PMI) plot of PKIs in clinical trials (yellow), approved (red) and from ChEMBL database (light blue).

The PMI plot (Figure 4) reveals a vast majority of kinase inhibitors are located along the rod-disc axis, indicating a preponderance of flat molecules, explained by the fact that all these molecules target a similar ATP active site. Indeed, most of the compounds in PKIDB are targeting the ATP site, thus, presenting a similar shape. Some of the MEK inhibitors are targeting an allosteric site adjacent to the ATP site. The three molecules from PKIDB closest to the extreme vertices are mubritinib near the rod, mavelertinib near the disc, and galunisertib (yellow dot in Figure 4) and idelalisib (red dot in Figure 4) near the sphere. They are all in clinical trials, in phase 1, 0, and 2, respectively. Unlike approved PKI, a few compounds in development tend to adopt a disc shape that explores a new molecular space in PKIs. We also observe some compounds from PKI_ChEMBL dataset getting closer to the sphere vertex, showing that some spherical molecules could also inhibit protein kinases. These ones could open the way to the exploration of a potential novel chemical space.

Here again, there is a great resemblance between the two datasets, PKIDB being well encompassed in PKI_ChEMBL regarding shape diversity.

### 2.3. Scaffold Diversity Assessment

#### 2.3.1. Analysis of Molecular Scaffolds

To get a deeper insight on the molecular diversity of PKIs, we focused on scaffolds and ring systems of these compounds. The results of scaffold analysis are summarized in Table 4. First, we looked for the presence of macrocyclic molecules (rings > 12 atoms). In PKIDB, there are four macrocycles. Two of them are approved drugs: icotinib, approved by CFDA and lorlatinib, and two are in phase 3: pacritinib and ruboxistaurin. This class of molecules might not be fully explored, since the percentage of macrocycles found in PKI_ChEMBL is very weak (<1%). As mentioned above, it is important to note that we excluded from PKIDB macrocycles containing the stem ‘imus’. However, these compounds do not directly target a kinase binding site, but rather an upstream protein, causing a complex formation that inhibits the kinase [32].

**Figure 5:**
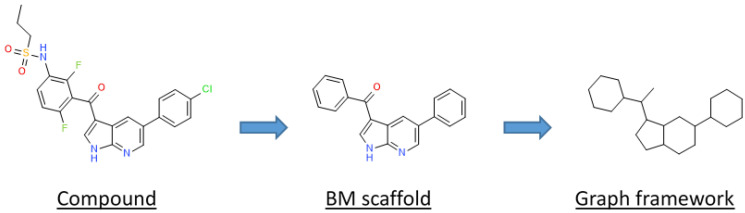
Representation of a molecular decomposition into scaffolds according to Bemis and Murcko (BM) and in graph framework.

The different types of molecular scaffolds are shown in Figure 5. For this study we used two types of scaffolds: Bemis and Murcko (BM) and a graph framework issued from BM. As a reminder, Bemis and Murcko scaffold corresponds to the core of a molecule after removing side chains [20]. The graph framework corresponds to BM scaffold, where each heteroatom by a carbon and each multiple bond was substituted by a single one. Therefore, such frameworks cover topologically equivalent BM scaffolds differentiated by heteroatom substitutions and bond types.

In PKIDB dataset, among 218 molecules, 207 present a unique BM scaffold and 195 a unique graph framework (GF). Whereas, for the 76,504 PKIs present in ChEMBL, only 28,732 and 13,331 BM scaffolds and GF, respectively, are found (Table 4). In other words, in PKIDB almost each compound has a unique scaffold (218/207). The pairwise molecular similarity means between PKIDB and PKI_ChEMBL, calculated with MACCS keys, indicates that both datasets are diverse, with a mean of Tanimoto similarity of about 0.5 (Table 4). However, in the PKI_ChEMBL dataset, the scaffold diversity corresponding to the total number of molecules over the total number of BM scaffolds, is much lower with about a BM scaffold for about 2.7 molecules in average. Regarding the graph frameworks, their number tends to decrease compared to BM scaffolds i.e., one GF for 1.1 and 5.7 molecules in PKIDB and PKI_ChEMBL, respectively.

**Figure 6:**
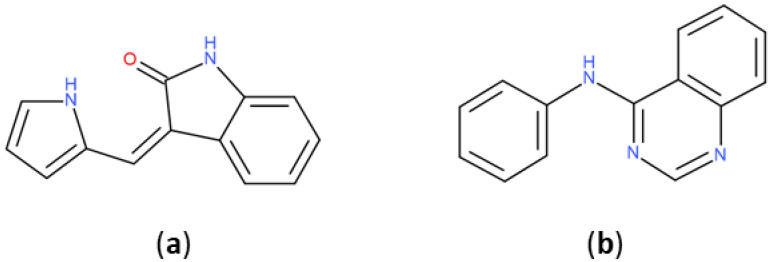
Most retrieved Bemis and Murcko scaffolds in PKIDB dataset (**a**): (3*Z*)-3-(1*H*-pyrrol-2-ylmethylene)indolin-2-one and in PKI_ChEMBL dataset (**b**): *N*-phenylquinazolin-4-amine.

The most represented BM scaffold in PKIDB, the indolinone derivative (Figure 6), is retrieved in three inhibitors and differs from the one in PKI_ChEMBL, which is found 644 times. This scaffold is prominent compared to others in PKI_ChEMBL: the second most retrieved scaffold, the quinazoline derivative, is only found 239 times. It shows the importance of that scaffold in PKIs, which is found only in erlotinib in PKIDB. The search for molecules containing PKIDB’s highest occurrence of BM scaffold in PKI_ ChEMBL only returns 10 compounds, revealing a major difference between the two datasets.

Then, for each unique BM scaffold in PKIDB, we checked how many PKIs are obtained in PKI_ChEMBL. From the 207 unique BM scaffolds available in PKIDB, only 107 are present in PKI_ChEMBL, which represent 2423 molecules out of a total of 76,504 (3.2%). This result is surprising. Firstly, we might expect that many analogues would be systematically provided for each PKI and, thus, would be available in a public database. Secondly, because PKIDB covers similar chemical space to PKI_ChEMBL according to PCA and PMI comparisons. Finally, using all unique graph frameworks from PKIDB, we were able to match 7686 compounds (10.0%) in PKI_ChEMBL.

#### 2.3.2. Ring Analysis

**Figure 7:**
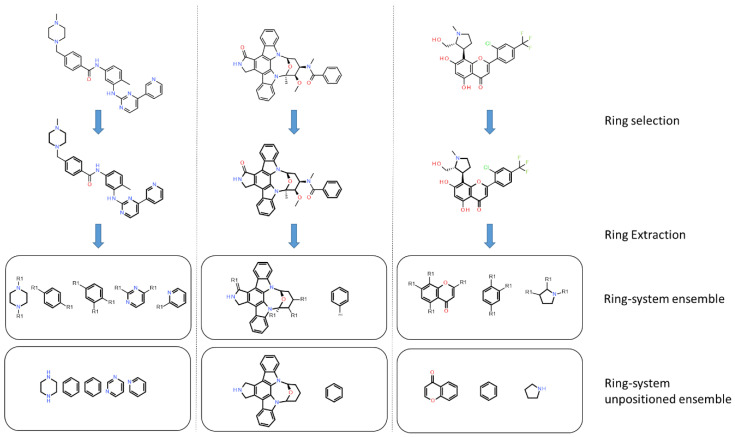
Application of the ring-system ensemble classification. Ring-system ensembles are obtained by removing substituents on acyclic bonds and by keeping attachment point (R1). The ring system unpositioned ensembles do not keep information on the attachment point. Rings are shown in bold.

In PKIs, rings are making hydrogen bonds, van der Waals or π-stacking interactions with residues of the active site. As example, an heterocycle may form hydrogen bonds, as does adenine in ATP with protein kinase [33]. We applied a molecular decomposition method, using RDKit to fragment molecules and retain only rings (Figure 7). After collecting all rings for both datasets, we searched for the most represented ones by gathering them using their smiles representation. We focused on fused heteroaromatic rings, since such fragments are present as a main scaffold in most kinase inhibitors. Moreover, fused rings offer favorable interactions (van der Waals and hydrogen bonds) into the ATP binding site compared to non-fused rings [34].

In both datasets, we found bicycles in around 65% of the molecules, demonstrating their importance as a core during hit to lead or lead optimization steps. In PKIDB, we found 56 unique bicyclic scaffolds among the total of 172. More surprising, 31 out of these 56 bicycles are singletons, i.e., the bicyclic scaffold is found only once in the dataset. For the PKI_ChEMBL dataset, there are 918 unique bicycles for a total of 57,438. However, among those 918 unique bicycles, only 26 are singletons. Since the PKI_ChEMBL dataset contains more analogues of chemical series compared to PKIDB, this could explain the lowest ratio of unique fused rings.

**Figure 8:**
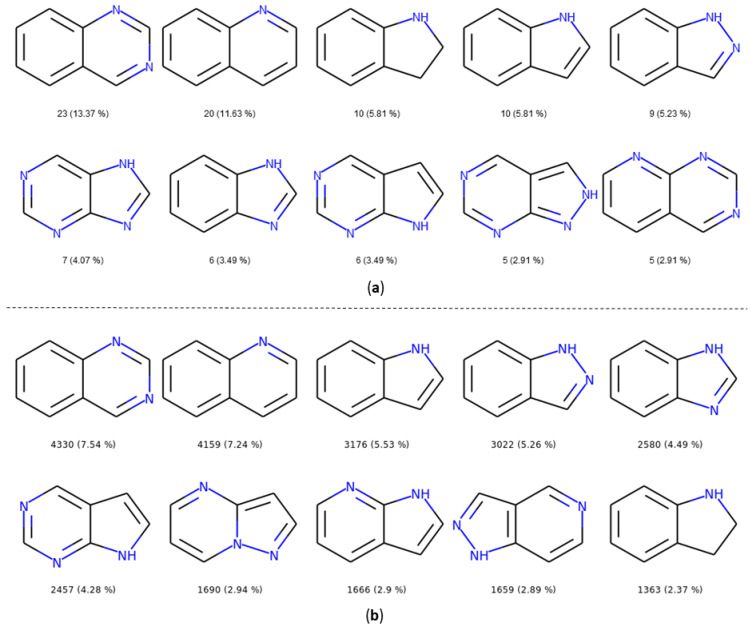
Top ten bicycles retrieved in the PKIDB dataset (**a**) and in PKI_ChEMBL (**b**) with their occurrence and their frequency in brackets. In PKIDB there are 172 bicycles (56 unique) and in PKI_ChEMBL, there are 57,439 bicycles (918 unique).

**Figure 9:**
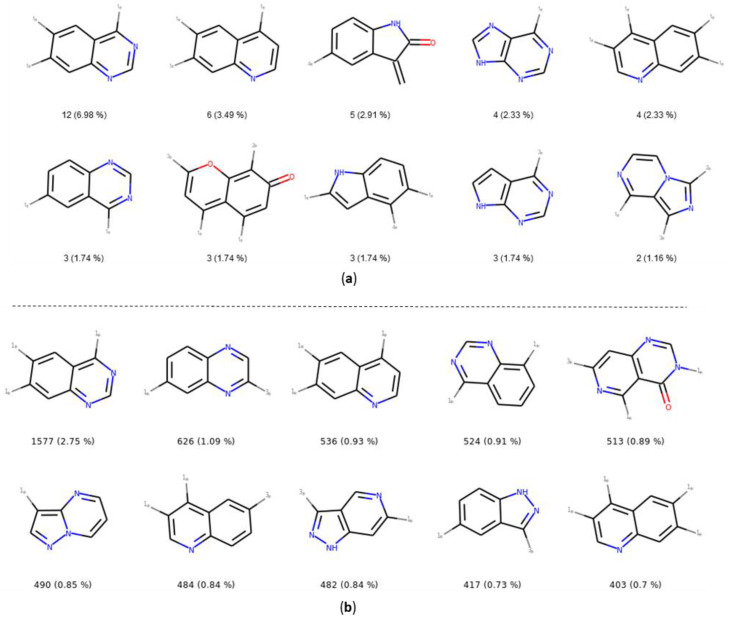
Top ten most retrieved bicycles with their substituents in the PKIDB dataset (**a**) and in PKI_ChEMBL (**b**) with their occurrence and their frequency in brackets. In PKIDB, there are 172 bicycles (129 unique) and in PKI_ChEMBL, there are 57,438 bicycles (4480 unique). 1_*_—connected to an atom not double bonded, not aromatic, not in a cycle and not halogen; 2_*_—connected to non aromatic ring; 3_*_—connected to aromatic atom; 4_*_—connected to an halogen.

The number and the frequency of the top 10 most retrieved bicycles are illustrated in Figure 8. In both datasets, the quinazoline scaffold is the most represented bicycle, it remains an important core, and its substituted analogues, such as the 4-anilinoquinazoline, have been extensively studied [35]. Examples of PKIs containing quinazoline scaffold are gefitinib, lapatinib, erlotinib, afatinib, and, more recently, canertinib [36]. Kinase inhibitors bearing this scaffold have mainly been designed to target EGFR. The second most represented bicyclic scaffold is the quinolone, another fused six-membered aromatic ring. It is worth noting that depending on the substituent types or the tautomeric form present in the molecules, a Rational Discovery Kit (RDKit) may break the aromaticity in the heterocyclic scaffolds. As an example, by removing the carbonyl functional group, considered as a substituent, in the indole scaffold, a non-aromatic indoline scaffold is kept, as shown in Figure 8. Most of the bicycles contain at least one heteroatom such as the nitrogen. This heteroatom allows H-bond interaction (acceptor or donor), with the hinge region of the kinase. Interestingly, the PKIDB and the PKI_ChEMBL datasets contain almost the same top ten bicyclic scaffolds. However, unlike BM scaffolds where more than half of them from PKIDB were not retrieved in PKI_ChEMBL, here, only three bicycles from PKIDB (not shown) are not retrieved in the PKI_ChEMBL dataset. We also performed an analysis of the bicyclic scaffolds by considering the attached atom position and atom type (Figure 9). Atoms involved in a double bond linked to the scaffold were not modified. However, all atoms were replaced by a dummy atom labelled differently according to the atom type (Figure 9). In this case, the 4,6,7-trisubstituted quinazoline is the most retrieved core in both datasets. Such a scaffold is found in twelve inhibitors in PKIDB, and an ether group (often a methoxy) is always attached on the 7 position. The second most retrieved bicycle is the 4,6,7-trisubstituted quinoline in PKIDB, and this is the third most represented scaffold in PKI_ChEMBL. Here again, the substituent in the 7 position is always an ether. Interestingly, the second most retrieved substituted bicycle in PKI_ChEMBL is not found in top tenth of PKIDB, as shown in Figure 8.

In Figure 9, the great majority of bicycles are polysubstituted, confirming their use as core scaffolds to link substituents. By considering the substituents during the analysis, the frequency of the bicycles shows a different distribution in both datasets, and the top ten bicyclic scaffolds are different.

## 3. Discussion

PKIDB is a freely available database containing all kinase inhibitors on the market or in clinical trials gathered using their international nonproprietary name (INN). This database, regularly updated, contains information on the structure of the kinase inhibitors, their physicochemical properties, their protein kinase targets, as well as their therapeutic indications. It also contains links to various external databases. We analyzed this dataset and compared it to active PKIs found in the ChEMBL database. Classical physicochemical descriptors, such as Lipinski’s or Veber’s, showed that a significant number of protein kinase inhibitors, either approved or in clinical trials, do not follow the recommended drug-like thresholds, especially regarding molecular weight and calculated LogP. Moreover, all PKI present in PKIDB violate a maximum of only two Lipinski’rules. Therefore, for this typical class of compounds, we propose new boundaries to better characterize the chemical space of kinase inhibitors. Moreover, all PKIS in PKIDB have a maximum of two chiral centers and five aromatic rings.

The projection of the chemical space resulting from a principal component analysis shows that most of the inhibitors shared the same chemical space. However, the PKIs available in ChEMBL fill a larger chemical space in the PCA plot compared to PKIs in PKIDB. The distribution of the physicochemical descriptors for both datasets do not present major differences. This suggests that most active PKIs available in the ChEMBL have drug-like properties.

Concerning the molecular shape of the PKIs, the PMI plot reveals that PKIs from ChEMBL exhibit a larger shape diversity compared to the ones in PKIDB. However, the majority of PKIs remain clustered around the rod-disc axis because they target a common ATP binding site in the kinase domain, which is highly conserved in this protein family. Yet, PKIs under development tend to explore wider topology, particularly near the disc edge. More frequent macrocyclic structures could contribute to this specific molecular shape. Moreover, moving to new chemical space will help medicinal chemists to escape from a crowded intellectual property (IP) space. Regarding PKIs in ChEMBL, we also found some compounds escaping from this rod-disc axis and get closer to the spherical form. This information could be used to design new chemically-diverse kinase inhibitors.

Concerning the molecular scaffold analysis of the two datasets, it appears that PKIs in PKIDB exhibit a great molecular scaffold diversity compared to the ones in ChEMBL. More than 100 scaffolds from PKIDB are not present in the ChEMBL. Each molecule present in PKIDB and, more particularly, the corresponding scaffold, was patented, preventing the design of analogues. Thus, each molecule present in PKIDB is in fact a representative of a chemical series, but only one new molecular entity (NME) was selected to continue its development in clinical phases. Most pharmaceutical companies will not unveil all chemical analogues of the selected NMEs, limiting information on the chemical series. On the opposite, in a public database such as ChEMBL, there are often lots of available analogues for a specific scaffold. The ring analysis performed on the two datasets indicates a similar number of bicycle singletons despite the large size difference in the two datasets, 218 vs. 76,504 molecules for PKIDB and PKI_ChEMBL, respectively. By considering the position and the type of the substituents, a significant part of the scaffolds in PKIDB are absent in ChEMBL, because most of the structures of pharmaceutical companies are protected by patents.

The PKIDB database is regularly updated and is accessible from this website: http://www.icoa.fr/pkidb. We hope that this resource will assist researchers in their quest for novel kinase inhibitors.

## 4. Materials and Methods

For the creation and maintenance of PKIDB please refer to our previous study [22]. All experiments and calculations have been performed with Python 3.6. Molecular descriptors used for PCA (Table 5) and PMI were calculated with RDKit (version ‘2018-09-01′, Palo Alto, CA, USA). Scaffolds analysis and clustering were performed with RDKIT and with Butina algorithm [37] using Tanimoto similarity and Morgan Fingerprint, with a radius of two (equivalent of FCPF4). The PCA was calculated with an in-house library derived from Prince [38] and Scikit-learn [39] packages (Rocquencourt, France). For PMI analysis, 3D conformations were generated using ETKDG method [40] followed with an energy minimization using the MMFF94 forcefield [41]. To delimit the dots of the PMI triangle, three compounds (diacetylene, benzene and adamantane) were considered and added to the dataset. All the figures are made using matplotlib [42] and seaborn [43] packages. Molecules were drawn with Biovia Draw 2018 (Velizy, France).

The PKI_ChEMBL dataset results from ChEMBL (version ‘ChEMBL_24′, Cambridgeshire, UK). To be included in this dataset, a compound must have at least one recorded activity, either IC_50_, Ki or Kd, on a protein kinase with a pchembl value > 6 (< 1000 nM). We then removed duplicates, empty SMILES and molecules from PKIDB. It is composed of 76,504 molecules. Both datasets (PKIDB and PKI_ChEMBL) have been prepared and standardized with VSPrep [44], and for each compound we kept the best tautomer as defined in VSPrep.

## Figures and Tables

**Table 1:** Protein kinase inhibitor (PKIs) approved in 2018 and 2019 with their respective targets extracted from DrugBank (Uniprot ID extracted from https://www.uniprot.org/).

PKI	Unitprot ID	Gene Name
Alpelisib	P42336	PI3KCA
Binimetinib	Q02750	MAP2K1
Dacomitinib	P00533	EGFR
Duvelisib	O00329	PI3KCD
	P48736	PI3KCG
Encorafenib	P15056	BRAF
Entrectinib	P04629	NTRK1
	Q16620	NTRK2
	Q16288	NTRK3
	P08922	ROS1
	Q9UM73	ALK
Erdafitinib	P11362	FGFR1
Fedratinib	O60674	JAK2
	P36888	FLT3
	O60885	BRD4
Fostamatinib	P43405	SYK
Gilteritinib	P36888	FLT3
	P30530	AXL
	Q9UM73	ALK
Larotractinib	P04629	NTRK1
	Q16620	NTRK2
	Q16288	NTRK3
Lorlatinib	Q9UM73	ALK
	P08922	ROS1
Pexidartinib	P36888	FLT3
	P10721	KIT
	P07333	CSF1R
Upadacitinib	P23458	JAK1
Zanubrutinib	Q06187	BTK

**Table 2:** Comparison of Lipinski’s rules violation between PKIs approved, in clinical trials and in ChEMBL.

^1^	0 Ro5 Violation	1 Ro5 Violation	2 Ro5 Violations	>2 Ro5 Violations
PKIs approved	33/60 (55.0%)	20/60 (33.0%)	7/60 (12.0%)	0/56 (0%)
PKIs in clinical trials	101/158 (64.0%)	41/158 (26.0%)	16/158 (10.0%)	0/158 (0%)
All PKIs	134/218 (61.5%)	61/218 (28.0%)	23/218 (10.5%)	0/218 (0%)
PKIs ChEMBL	51,858/76,504 (67.8%)	18,601/76,504 (24.3%)	5876/76,504 (7.7%)	169/76,504 (0.2%)

^1^ RDKit was used to calculate all descriptors including ClogP.

**Table 3:** Number of PKIs violating at least one Lipinski’s or Veber’s rule.

^1^	MW > 500 Da	ClogP > 5	HBA > 10	HBD > 5	TPSA > 140 Å^2^	NRB > 10
PKIs approved	20/60 (33.3%)	12/60 (20.0%)	2/60 (3.3%)	0/60 (0%)	2/60 (3.3%)	2/60 (3.3%)
PKIs in clinical trials	46/158 (29.1%)	26/158 (16.5%)	1/158 (0.6%)	0/158 (0%)	4/158 (2.5%)	6/158 (3.8%)
All PKIs	66/218 (30.3%)	38/218 (17.4%)	3/218 (1.4%)	0/218 (0%)	6/218 (2.8%)	8/218 (3.7%)
PKIs ChEMBL	18,892/76,504 (24.7%)	10,897/76,504 (14.2%)	924/76,504 (1.2%)	208/76,504 (0.2%)	3695/76,504 (4.8%)	2051/76,504 (2.7%)

^1^ RDKit was used to calculate all descriptors including ClogP.

**Table 4:** Data obtained for the Bemis and Murcko scaffolds for the two datasets.

	No. Molecules	No. Macrocycles	No. BM Scaffolds	No. Graph Frameworks	Molecular Similarity Mean ^a^ (SD)
PKIDB	218	4 (1.8%)	207 (95.0%)	195 (89.5%)	0.51 (0.11)
PKI_ChEMBL	76,504	487 (0.64%)	28,732 (37.6%)	13,331 (17.4%)	0.49 (0.11)

^a^ Calculated with MACCS keys (166 bits) and the Tanimoto coefficient.

**Table 5:** Descriptors used for PCA.

Name Variable	Descriptor
MW	Molecular weight
LogP	Wildman-Crippen LogP value
TPSA	Topological polar surface area
HBA	Number of Hydrogen Bond Acceptors
HBD	Number of Hydrogen Bond Donors
NRB	Number of Rotatable Bonds
NAR	Number of aromatic rings
FCSP3	Fraction of C atoms that are SP3 hybridized
MQN8	Molecular Quantum Numbers
MQN10	Molecular Quantum Numbers
